# A Novel Protocol to Accelerate Functional Orthopedic Treatment Using Low-Intensity Pulsed Ultrasound in Patients With Skeletal Class II Malocclusion: A Preliminary Clinical Report

**DOI:** 10.7759/cureus.105067

**Published:** 2026-03-11

**Authors:** Dima M. Almrayati, Mohammad Y. Hajeer, Ahmad S. Burhan, Wael H. Almahdi, Samer T. Jaber

**Affiliations:** 1 Department of Orthodontics, Faculty of Dentistry, University of Damascus, Damascus, SYR; 2 Department of Periodontics, Faculty of Dentistry, University of Damascus, Damascus, SYR; 3 Department of Orthodontics, Faculty of Dentistry, Al-Wataniya Private University, Hama, SYR

**Keywords:** activator, bone remodeling, class ii malocclusion, dental and skeletal changes, functional treatment, low intensity pulsed ultrasound, mandibular advancement, orthopedic correction

## Abstract

Background and objective: This preliminary study aimed to evaluate the effectiveness of a novel, clinically feasible low-intensity pulsed ultrasound (LIPUS) protocol in accelerating functional orthodontic treatment using removable appliances.

Methods: Five patients (mean age: 11.10 ± 0.41 years) diagnosed with Class II Division 1 malocclusion underwent functional orthodontic treatment supplemented with the LIPUS, US PRO 2000™ 2nd Edition device (Current Solutions, Austin, Texas, United States). Ultrasound stimulation was applied twice weekly during the first month, once every two weeks during the second month, and once every three weeks thereafter until completion of active treatment. The primary outcomes assessed were the duration of active treatment and dentoskeletal changes.

Results: The mean duration of active treatment was 170.80 ± 19.76 days. Significant sagittal skeletal improvements were observed, including forward mandibular repositioning, reflected by an increase in the SNB angle "the Sella-Nasion plane to Point B angle" (mean difference (MD) = +1.32°), accompanied by a slight reduction in the SNA angle "the Sella-Nasion plane to Point A angle" (MD = −0.54°), a decrease in the ANB angle "the difference between SNA and SNB angles" (MD = −1.98°), an increase in the SNPog angle "the Sella-Nasion plane to Point Pog angle" (MD = +0.94°) further confirmed anterior mandibular displacement. Vertical skeletal parameters remained largely stable, with no evidence of adverse vertical effects. Dentoalveolar changes included controlled retroclination of the maxillary incisors, proclination of the mandibular incisors, and clinically relevant reductions in overjet (MD = −4.38 mm) and overbite (MD = −1.06 mm).

Conclusions: The modified LIPUS protocol produced favorable skeletal and dentoalveolar effects while maintaining vertical stability, suggesting it may represent a practical alternative to conventional daily ultrasound application during functional orthodontic treatment.

## Introduction

Class II malocclusion represents one of the most prevalent skeletal discrepancies encountered in orthodontic practice. The choice of an appropriate treatment strategy depends on several factors, including the patient’s chronological and skeletal age, the underlying etiologic pattern, and the degree of maxillomandibular disharmony. Conventional management approaches encompass growth modification, dental camouflage, and orthognathic surgery [1].

Among these, functional treatment remains the cornerstone for growing patients, as mandibular deficiency is commonly implicated in the etiology of Class II malocclusion [2]. This modality aims to stimulate mandibular growth and reestablish craniofacial balance [3]. However, the success of removable functional appliances largely depends on patient compliance with the prescribed wear time [4,5]. Prolonged treatment durations often compromise motivation and adherence, potentially limiting the effectiveness of growth modification during the optimal developmental period [4].

To overcome these limitations, recent advances in orthodontics have focused on accelerating tooth movement and enhancing skeletal adaptation to reduce overall treatment duration while maintaining biological safety [6-8]. Various approaches have been explored, including biological [9,10], surgical [11,12], mechanical [13,14], and physical acceleration methods [15]. Among these, physical stimulation techniques, such as low-level laser therapy [16], vibration [17], and low-intensity pulsed ultrasound (LIPUS) [17], have gained increasing attention due to their noninvasive nature and ability to modulate cellular activity and tissue remodeling. These modalities aim to enhance bone turnover and promote faster orthodontic or orthopedic responses without compromising treatment outcomes or patient comfort [18].

Specifically, LIPUS has emerged as one of the most promising physical modalities. Initial in vitro investigations exploring the biological potential of LIPUS had demonstrated that it can stimulate cellular activity, accelerating bone and cartilage cell proliferation and regeneration [19,20]. Subsequent animal studies further substantiated these findings, providing further evidence that LIPUS enhances osteogenesis and chondrogenesis by promoting tissue remodeling and accelerating skeletal development [21].

Namera et al. conducted the first orthodontic investigation to explore the potential of ultrasound as an adjunct to functional orthopedic treatment in orthodontics [22]. In their study, bilateral ultrasound stimulation was applied without direct skin contact, using a frequency of 1 MHz and an intensity of 0.03 W/cm². The application was performed in continuous circular motion for 20 minutes per side daily for the initial 21 days, followed by maintenance sessions once every three weeks until completion of active therapy. The authors reported a substantial 44% reduction in total treatment duration, highlighting the potential of ultrasound to enhance the efficiency of functional appliances. However, the protocol was criticized for its clinical impracticality, as the intensive daily application during the first 21 days was difficult to implement in a routine orthodontic setting. This limitation emphasized the necessity for a clinically practical, patient-friendly LIPUS protocol suitable for routine orthodontic application.

In light of these findings, the Department of Orthodontics at Damascus University, Syria, developed a novel, clinically feasible LIPUS protocol to overcome the practical limitations observed in previous applications while preserving patient comfort and treatment safety. This innovative protocol was carefully structured to optimize both the frequency and duration of ultrasound exposure, ensuring adequate biological stimulation without imposing excessive clinical or compliance demands. The approach was specifically tailored to complement functional orthopedic therapy in growing Class II patients, with the aim of accelerating mandibular growth response and improving treatment efficiency within a realistic clinical setting.

## Materials and methods

Study design and settings

This preliminary clinical experimental study assessed the feasibility and efficacy of a novel, clinically applicable LIPUS protocol intended to accelerate functional therapy. Performed at the Department of Orthodontics, Faculty of Dentistry, Damascus University between September 2022 and March 2023, the investigation was approved by the Medical Research Ethics Council of Damascus University (serial number: DN‑30082022‑10) and functioned as a pilot phase to inform a planned randomized controlled trial (RCT) comparing LIPUS with low‑level laser therapy for accelerated bone remodeling following functional orthopedic treatment; the pilot was undertaken to validate the proposed LIPUS application method. Because its purpose was methodological refinement rather than hypothesis testing in a full RCT, the study was not registered in a clinical trials registry

Study population

Participants were recruited from the pending treatment records at Damascus University. The inclusion and exclusion criteria used for patient selection are presented in Table 1. After confirming eligibility, the study protocol and objectives were explained to participants’ parents or legal guardians, and written informed consent was obtained

**Table 1:** Inclusion and exclusion criteria for patient selection. ANB: difference between SNA angle (Sella–Nasion–Point A) and SNB angle (Sella–Nasion–Point B); TMJ: temporomandibular joint.+

Inclusion criteria	Exclusion Criteria
Skeletal Class II Division 1 malocclusion with an ANB angle greater than 5°	Previous orthodontic treatment
Overjet > 4 mm	Craniofacial anomalies
Normal inclination of lower incisors	TMJ disorders
Horizontal or average facial growth pattern	Systemic diseases affecting bone metabolism
Evidence of being in a pubertal growth spurt using hand-wrist radiographs.	

Functional appliance and acceleratory intervention

**Figure 1:**
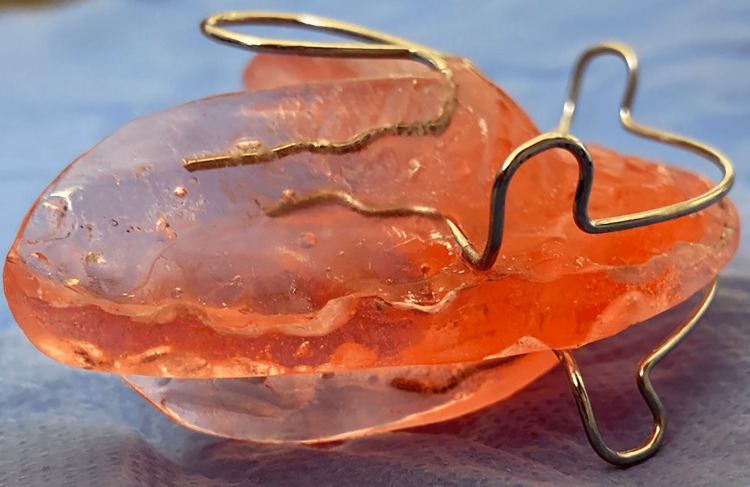
The design of the Activator appliance that was used in the current study

All appliances were fabricated by the same orthodontic technician using self-curing acrylic to ensure consistency. The Modified Activator employed in this study was based on the design introduced by Schmuth in 1971 [23]. It featured an acrylic body with two splints meeting at the occlusal plane, upper and lower labial bows, a Coffin Spring in the upper plate, and an acrylic cap to retain the lower incisors (Figure 1). Each subject underwent a single-phase mandibular advancement to achieve an edge-to-edge incisal relationship, with a vertical opening of 5-6 mm in the premolar region. Patients were instructed to wear the appliance for 18 hours daily, with parental supervision to ensure compliance.

**Figure 2:**
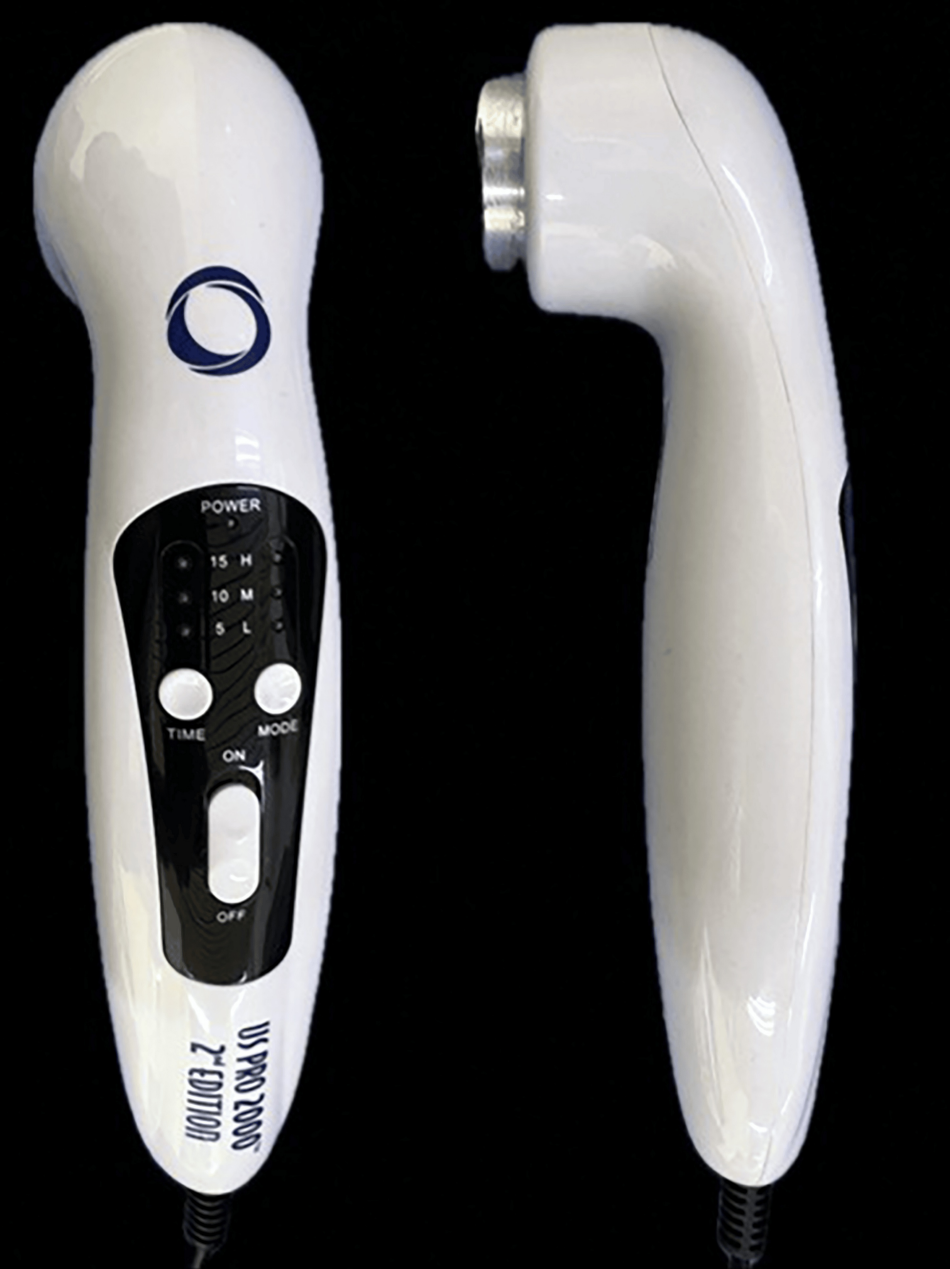
The low-intensity pulsed ultrasound (LIPUS) device that was used in the current study.

LIPUS was administered using the US PRO 2000™ 2nd Edition device (Current Solutions, Austin, Texas, United States) (Figure 2). A conductive gel was applied to the right and left temporomandibular joint (TMJ) regions to facilitate wave transmission. The device emitted 200-µs bursts of a 1-MHz sine wave at 100 Hz, with a temporal-average intensity of 0.03 W/cm². LIPUS treatment was delivered twice weekly during the first month, once every two weeks in the second month, and once every three weeks thereafter until the completion of active treatment. A single researcher administered all treatments. During follow-up visits, the occlusal bite plane was progressively reduced to facilitate posterior tooth eruption, promote occlusal settling, and assist in levelling the occlusal plane.

Primary outcome measure: active treatment time

The duration of active treatment was assessed by measuring the interval, in days, between baseline (T0) and the end of active treatment (T1), which was defined by the achievement of molar Class I, overjet ≤3 mm, stable chin position under digital pressure, and normal overbit.

Secondary outcome measures

**Figure 3:**
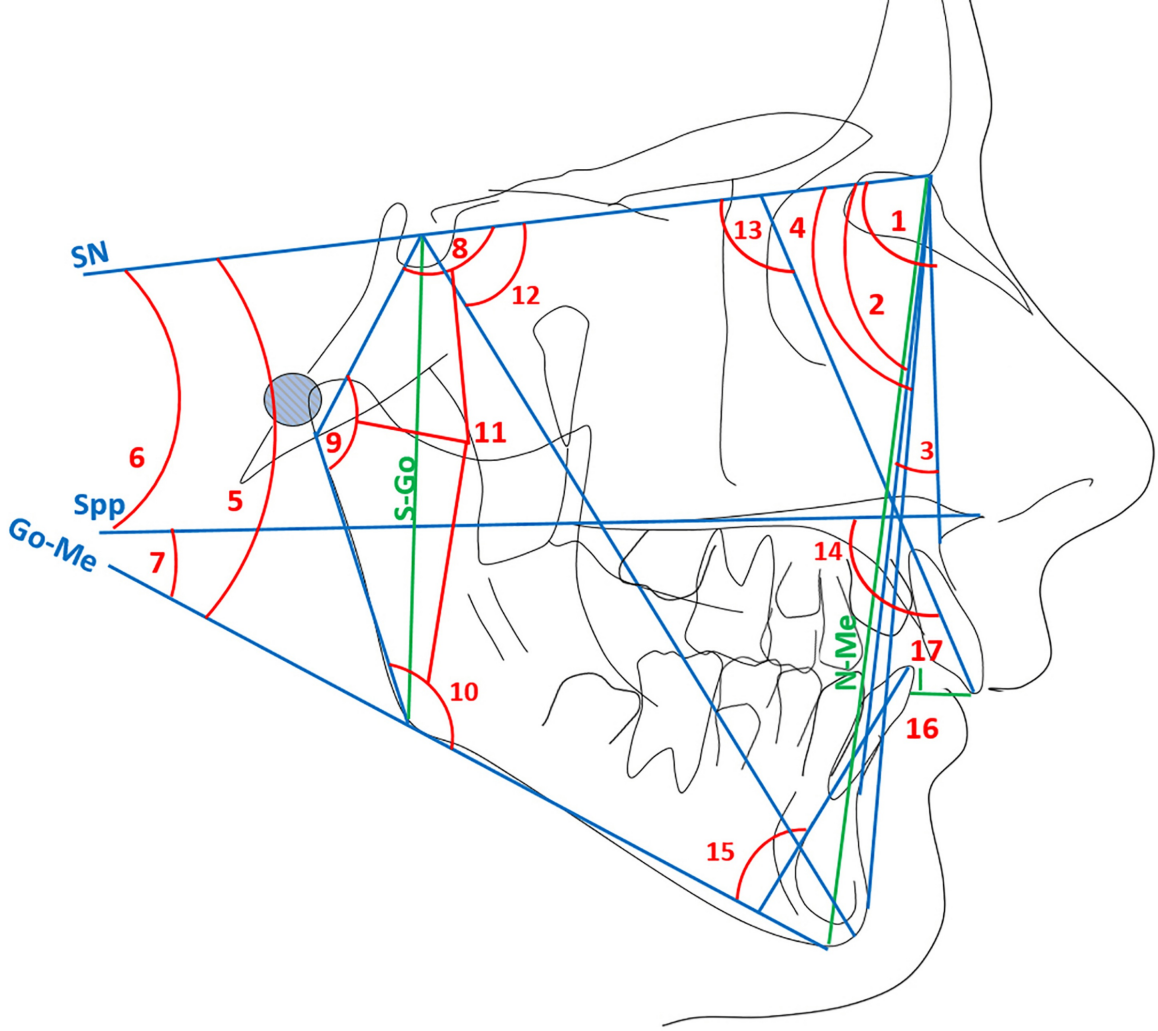
Cephalometric measurements made on the tracings of the radiographs. 1: SNA: the angle between the anterior cranial base and the NA plane; 2: SNB : the angle between the anterior cranial base and the NB plane;  3: ANB : the angle between the NA plane and the NB plane;  4: SNPog: the angle between the anterior cranial base and the Nasion–Pogonion (NPog) plane; 5:SN-GoMe: the angle between the anterior cranial base and the mandibular plane; 6: SN-Spp: the angle between the anterior cranial base and the maxillary plane; 7: MM : the angle between the maxillary plane (Spp plane) and the mandibular plane (GoMe plane);  8: NSAr: the angle between N, S, and Ar points (the interior angle);  9: SArGo: the angle between S, Ar, and Go points (the interior angle); 10: ArGoMe: angle between Ar, Go, and Me points (the interior angle); 11: Bjork's sum : the sum of NSAr, SArGo, and ArGoMe angles; 12: Y-axis: the angle between the anterior cranial base and the Y-axis (S-Gn) point; 13:U1-SN: the angle between the anterior cranial base and the upper incisor axis; 14:U1-Spp: the angle between the maxillary plane (SPP plane) and the upper incisor axis; 15: L1-GoMe: The angle between the mandibular plane and the lower incisor axis; 16: Overjet: the horizontal overlap between the upper and lower incisors; 17: Overbite: the vertical overlap between the upper and lower incisors. Image created by authors using Microsoft Publisher Version 16 (Microsoft Corporation, Redmond, Washington, United States)

The secondary outcomes of this study were the skeletal and dentoalveolar changes observed following completion of the active phase of functional appliance therapy. These changes were evaluated using lateral cephalometric radiographs obtained at baseline (T0) and at the end of active treatment (T1). Cephalometric tracings and measurements were performed using the Viewbox® software (version 4.0.0.98; dHAL Software, Kifissia, Greece). All landmarks were identified and measured twice by a single trained examiner to ensure reliability. Linear and angular differences between T0 and T1 were extracted for subsequent analysis. The cephalometric measurements are illustrated in Figure 3, and their definitions are detailed in the Appendices. The definitions were derived from Jacobson [24] and Riolo [25]. Linear measurements were corrected for radiographic magnification, as detailed in the Appendices.

Statistical analysis

All statistical analyses were performed using the IBM SPSS Statistics for Windows, version 22.0 (IBM Corp., Armonk, New York, United States). Descriptive statistics, including mean, median, minimum, and maximum values, were computed for all participants. Treatment effects were quantified as the mean differences between T0 and T1 measurements.

## Results

Baseline sample characteristics

After obtaining their consent, five patients (four female and one male), with a mean age of 11.10 ± 0.41 years, were selected and enrolled in this preliminary study. After the follow-up period, the data from these five patients were analyzed. The patients' pre-treatment characteristics are presented in Table 2.

**Table 2:** Baseline characteristics of the sample at the beginning of the treatment (N=5) SD: standard deviation; SNA:  the angle between the anterior cranial base (SN) and the NA plane; SNB: the angle between the anterior cranial base (SN) and the NB plane; ANB: the angle between the NA plane and the NB plane; SN-GoMe: the angle between the anterior cranial base (SN) and the mandibular plane (GoMe); L1-GoMe: The angle between the mandibular plane and the lower incisor axis (L1).

Parameter	Value
Age (years), mean ± SD	11.10 ± 0.41 years
Sex, male/ female	1/4
Overjet, mean ± SD	7.48± 1.31 mm
SNA, mean ± SD	80.60 ±1.83°
SNB, mean ± SD	74.20 ±2.29°
ANB, mean ± SD	6.40 ±1.08°
SN-GoMe, mean ± SD	34.26 ± 2.61°
L1-GoMe, mean ± SD	93.50 ± 1.87°

Overall duration of active treatment

The mean treatment duration was 170.80 ± 19.76 days, ranging from 150 to 200 days, with a median of 174.50 days. More details are presented in Table 3.

**Table 3:** Descriptive statistics of the cephalometric variables before and after treatment SNA: the angle between the anterior cranial base and the NA plane; SNB : the angle between the anterior cranial base and the NB plane; ANB : the angle between the NA plane and the NB plane; SNPog: the angle between the anterior cranial base and the Nasion–Pogonion (NPog) plane; SN-GoMe: the angle between the anterior cranial base and the mandibular plane; SN-Spp: the angle between the anterior cranial base and the maxillary plane; MM : the angle between the maxillary plane (Spp plane) and the mandibular plane (GoMe plane); NSAr: the angle between N, S, and Ar points (the interior angle); SArGo: the angle between S, Ar, and Go points (the interior angle); ArGoMe: angle between Ar, Go, and Me points (the interior angle); Bjork's sum: the sum of NSAr, SArGo, and ArGoMe angles; Y-axis: the angle between the anterior cranial base and the Y-axis (S-Gn) point; U1-SN: the angle between the anterior cranial base and the upper incisor axis; U1-Spp: the angle between the maxillary plane (SPP plane) and the upper incisor axis; L1-GoMe: The angle between the mandibular plane and the lower incisor axis; Overjet: the horizontal overlap between the upper and lower incisors; Overbite: the vertical overlap between the upper and lower incisors;  Jarabak's ratio: the ratio between the posterior facial height (measured from S to Go) and the anterior facial height (measured from N to Me); T0: baseline; T1: at the end of the active treatment.

Variable	Mean ± SD			Median		Minimum		Maximum	
	T0	T1	Mean difference	T0	T1	T0	T1	T0	T1
SNA (°)	80.60 ±1.83	80.06 ±2.06	-0.54	80.30	79.40	79	77.60	83	83.10
SNB (°)	74.20 ±2.29	75.52 ±2.33	+1.32	74.30	75.60	71.20	72.60	77.60	79.00
ANB (°)	6.40 ±1.08	4.42 ±0.75	-1.98	6.80	4.30	5.00	3.40	7.40	5.30
SNPog (°)	75.54 ±2.68	76.48 ±2.29	+0.94	75.50	75.90	72.50	73.90	79.80	80.10
SN-Spp (°)	7.78 ± 1.01	7.82± 1.04	+0.04	7.90	7.90	6.30	6.70	9.10	9.20
SN-GoMe (°)	34.26 ± 2.61	33.74 ± 2.30	-0.52	34.10	33.20	31.40	31.10	37.10	37.40
MM (°)	26.34 ± 2.78	27.30 ± 2.52	+0.96	26.80	27.10	22.50	23.50	29.00	30.10
NSAr (°)	126.68 ± 4.42	126.74 ± 4.24	+0.06	126.10	127.30	121.10	120.30	131.10	131.90
SarGo (°)	144.4 ± 4.78	144.96 ± 7.41	+0.56	143.30	144.10	139.10	138.30	151.90	156.70
ArGoMe (°)	124.12 ± 5.26	123.72± 5.13	-0.40	124.50	123.50	117.80	117.50	131.80	129.60
Bjork (°)	396.60 ± 2.80	395.42 ± 3.12	-1.18	395.50	394.50	392.60	392.40	399.20	400.50
Y-axis (°)	68.36 ± 0.46	69.44± 1.47	+1.08	68.10	68.90	68.00	68.30	69.00	72.00
U1-SN (°)	102.04 ± 6.70	99.60 ± 6.05	-2.44	100.70	97.00	94.90	93.30	112.60	108.50
U1-Spp (°)	112.30 ± 2.12	107.20 ± 6.87	-5.10	113.00	102.70	109.00	101.60	114	116.00
L1-GoMe (°)	93.50 ± 1.87	101.10 ± 2.60	+7.90	94.00	104.00	92.54	105.20	95.00	108.40
Jarabak (%)	65.80 ± 2.68	65.34 ± 3.25	-0.46	66.00	66.70	63.00	61.00	70.00	69.00
Overjet (mm)	7.48 ± 1.31	3.10 ± 0.54	-4.38	7.30	3.50	6.20	3.10	9.70	4.50
Overbite (mm)	4.58 ± 0.59	3.52 ± 0.76	-1.06	4.50	3.70	3.90	2.20	5.50	4.10

Sagittal skeletal changes

Substantial sagittal skeletal changes were evident throughout the course of treatment (Table 2). The mandible demonstrated a clear forward positional response, reflected by an increase in the Sella-Nasion to Point B angle "SNB angle" (mean difference (MD) = +1.32°). This was accompanied by a slight reduction in the Sella-Nasion to Point A angle "SNA angle" (MD = -0.54°). Consequently, the maxillomandibular skeletal relationship showed a marked improvement, as indicated by the decrease in the angle of difference between SNA and SNB "ANB angle" (MD = -1.98°). Furthermore, the Sella-Nasion to Point Pog angle "SNPog angle" increased (MD = +0.94°), suggesting anterior repositioning of the pogonion relative to the cranial base and reinforcing the favorable skeletal response to treatment.

Vertical skeletal changes

Vertical skeletal parameters remained largely stable throughout treatment (Table 2), with only minimal adjustments observed. The maxillary plane inclination (SN-Spp), the angle between the Sella-Nasion plane and the maxillary plane "Spp", exhibited a negligible increase (MD = +0.04°), whereas the mandibular plane angle (SN-GoMe), the angle between the Sella-Nasion plane and the mandibular plane Gonion-Menton plane, showed a slight decrease (MD = -0.52°), indicating maintenance of the vertical maxillomandibular relationship. The intermaxillary angle (MM), the angle between the maxillary plane "Spp plane" and the mandibular plane "GoMe plane", demonstrated a modest increase (MD = +0.96°), and the Y-axis, the angle between Sella-Nasion to Gnathion point, presented a mild forward-downward rotational tendency (MD = +1.08°). Additionally, a small reduction in Jarabak's ratio (MD = -0.46%) was associated with a subtle increase in lower anterior facial height. Overall, these vertical skeletal modifications represent minor adaptive changes, with no evidence of adverse vertical changes.

Dental changes

Pronounced dentoalveolar modifications accompanied the skeletal changes observed during treatment (Table 2). The maxillary incisors demonstrated controlled retroclination, evidenced by reductions in both U1-SN, the angle between the anterior cranial base Sella-Nasion and the upper incisor axis (MD = -2.44°), and U1-Spp, the angle between the maxillary plane SPP plane and the upper incisor axis (MD = -5.10°). In contrast, the mandibular incisors exhibited substantial proclination, with an increase in L1-GoMe, the angle between the mandibular plane and the lower incisor axis (MD = +7.90°), reflecting a typical dental adaptation associated with functional appliance therapy. Additionally, clinically relevant improvements were noted in overjet and overbite, which decreased by a mean of 4.38 mm and 1.06 mm, respectively, indicating significant sagittal and vertical dentoalveolar compensation.

## Discussion

The primary aim of this preliminary study was to assess the therapeutic effectiveness of a newly proposed LIPUS protocol as a substitute for the conventional 21-day continuous daily application, and to determine whether this modified approach could improve treatment efficiency and potentially shorten the duration required for functional orthopedic correction. LIPUS represents a non-invasive adjunctive modality known to stimulate cellular activity, accelerate bone remodeling, and enhance skeletal adaptation during growth modification therapy [26].

A notable innovation introduced by the present study is the implementation of a revised LIPUS application schedule specifically designed to lessen the overall treatment burden without compromising clinical efficacy. In contrast to the traditional regimen, requiring uninterrupted daily use over three consecutive weeks and commonly associated with reduced patient compliance and increased clinical follow-up requirements, the alternative protocol investigated here is more time-efficient and practically easier to integrate into routine care [22]. This adjustment carries the potential to improve patient cooperation, reduce cumulative exposure, and streamline clinical workflow while still achieving meaningful skeletal and dentoalveolar response.

Regarding treatment efficiency, an earlier study using the conventional LIPUS protocol reported an active treatment duration of approximately 160 days [22]. In comparison, the modified protocol used in the present study achieved similar skeletal and dentoalveolar effects within a mean duration of 170 days. While this small 10-day difference is not clinically significant, it indicates that the modified protocol maintains treatment effectiveness while offering practical advantages and good patient compliance.

The results of this preliminary study demonstrate that the modified LIPUS protocol effectively elicited favorable skeletal and dentoalveolar adaptations. On the sagittal plane, all patients exhibited forward mandibular repositioning, as evidenced by an increase in the SNB angle (mean of +1.32°) and a corresponding reduction in the ANB angle (mean of -1.98°). The increase in the SNPog angle (mean of +0.94°) further supports true anterior movement of the Pogonion point relative to the cranial base, indicating that mandibular advancement was achieved rather than being solely a dentoalveolar compensation. These changes translated clinically into improved maxillomandibular relationships and a significant reduction in sagittal discrepancy, confirming that the modified protocol can effectively stimulate mandibular growth response during functional treatment.

When contextualizing our findings within the existing literature, the meta-analytic data reported by Koretsi et al. indicate that conventional Activator therapy yields relatively modest skeletal changes, with a mean decrease of 0.28° in the SNA angle, a mean increase of 0.62° in the SNB angle, and a reduction (mean of 1.14°) in the ANB angle [27]. In comparison, our study demonstrated more substantial skeletal responses, particularly with greater mandibular advancement and a more marked improvement in the maxillomandibular relationship. These observations suggest that the modified protocol employed in this investigation may provide enhanced sagittal skeletal correction, highlighting its potential to achieve more clinically significant maxillofacial adaptations than those typically reported for standard Activator therapy.

Moreover, a previous study that applied the conventional daily 21-day ultrasound exposure protocol reported even greater increases in the SNB angle and more pronounced decreases in the SNA and ANB angles [22]. Nevertheless, despite these differences in numerical values, the variations between the outcomes of the present study and the previous investigation are not considered clinically significant, indicating that the modified protocol still achieves meaningful maxillomandibular correction while offering advantages in treatment practicality and patient compliance.

Vertical skeletal parameters remained largely stable throughout the treatment period. Minor adjustments, including a slight decrease in the mandibular plane angle (SN-GoMe, mean of -0.52°) and a small increase in the Y-axis angle (mean of +1.08°), indicate that the vertical dimension was maintained, with no evidence of adverse clockwise rotation or excessive lower anterior facial height. Preservation of vertical stability is particularly important in growing patients, as it prevents unintended alterations to facial proportions while allowing sagittal correction to proceed efficiently. These findings are consistent with those reported by Namera et al., who also observed minimal changes in vertical skeletal parameters during functional appliance therapy, reinforcing the reliability of this approach in maintaining vertical facial relationships [22].

Dentoalveolar changes accompanied the skeletal improvements, contributing to functional correction without over-reliance on dental compensation. Maxillary incisors underwent controlled retroclination (U1-SN: mean of -2.44°, U1-Spp: mean of -5.10°), whereas mandibular incisors exhibited marked proclination (L1-GoMe: mean of +7.90°), reflecting the expected adaptive response to functional therapy. Clinically relevant improvements in overjet (mean of -4.38 mm) and overbite (mean of -1.06 mm) further corroborate the combined skeletal and dental effects, demonstrating that the modified LIPUS schedule supports both structural and occlusal correction within a shortened treatment timeframe. Notably, these dentoalveolar changes are consistent with those reported in the earlier study using the conventional protocol, indicating that the modified application did not alter the characteristic dental response typically associated with functional appliance therapy [22].

Overall, these findings suggest that the modified, less time-intensive LIPUS protocol successfully stimulates targeted skeletal remodeling and dentoalveolar adaptation while maintaining vertical stability. This provides preliminary evidence that the new protocol may reduce treatment duration and patient burden without compromising clinical outcomes, highlighting its potential as an effective alternative to the traditional daily 21-day regimen.

Limitations and future recommendations

Although this is the first study to evaluate the latest LIPUS protocol, it has several limitations. The absence of a control group and the small sample size were primary limitations. Future research should incorporate randomized controlled designs with larger, diverse populations and extended follow-up periods to comprehensively evaluate the effects of the LIPUS protocol on stability, TMJ changes, and patient-reported outcomes.

## Conclusions

Within the limitations of this preliminary study, the modified LIPUS application protocol appears to be effective in enhancing sagittal skeletal correction during functional orthodontic treatment while maintaining vertical skeletal stability. The protocol achieved favorable skeletal and dentoalveolar outcomes within a treatment duration comparable to conventional regimens, suggesting that it may reduce treatment burden and improve clinical practicality without compromising therapeutic effectiveness. Further studies with larger sample sizes and controlled designs are warranted to confirm these findings.

## Appendices

**Table 4:** Definitions of the angular and linear measurements used in this study Variable definitions were adopted from Jacobson, 1995 [24] and Riolo, 1974 [25].

Variable	Definition
SNA	The angle between the anterior cranial base and the NA plane.
SNB	The angle between the anterior cranial base and the NB plane.
ANB	The angle between the NA plane and the NB plane.
SNPog	The angle between the anterior cranial base and the Nasion–Pogonion (NPog) plane.
SN-Spp	The angle between the anterior cranial base and the maxillary plane.
SN-GoMe	The angle between the anterior cranial base and the mandibular plane.
NSAr	The angle between N, S, and Ar points (the interior angle).
SArGo	The angle between S, Ar, and Go points (the interior angle).
ArGoMe	The angle between Ar, Go, and Me points (the interior angle).
Björk's sum	The sum of NSAr, SArGo, and ArGoMe angles.
Y-axis	The angle between the anterior cranial base and the Y-axis.
MM	The angle between the maxillary plane (Spp plane) and the mandibular plane (GoMe plane).
U1-SN	The angle between the anterior cranial base and the upper incisor axis.
U1-Spp	The angle between the maxillary plane (SPP plane) and the upper incisor axis.
L1-GoMe	The angle between the mandibular plane and the lower incisor axis.
Jarabak's ratio	The ratio between the posterior facial height (measured from S to Go) and the anterior facial height (measured from N to Me).
Overjet	The horizontal overlap between the upper and lower incisors.
Overbite	The vertical overlap between the upper and lower incisors.
